# Exploring supply and demand imbalance of community-based older adult care: an observational study in Chongqing, China

**DOI:** 10.3389/fpubh.2025.1581039

**Published:** 2025-06-11

**Authors:** Yilin Zhou, Xiaoqin Tian, Yuqin Tang, Peng Peng, Dan Deng

**Affiliations:** Department of Health Statistics, College of Public Health, Chongqing Medical University, Chongqing, China

**Keywords:** community-based older adult care, Kano model, analytic hierarchy process, cluster analysis, supply and demand

## Abstract

**Objective:**

Using an urban area in southwest China as a case study, this research investigates and analyzes the supply and demand of community-based older adult care services to provide a basis for optimizing the older adult care service system.

**Methods:**

A two-stage sampling method was employed to select older participants. Questionnaires, designed based on the Kano model, were administered to participants, and semi-structured interviews were conducted with community senior center managers and services users. The Kano model, Better-Worse index analysis and Analytic Hierarchy Process (Kano-BW-AHP) was used for quantitative analysis, while the two-step cluster analysis was employed for user profiles. Qualitative analysis was based on interview data.

**Results:**

A total of 259 questionnaires and 16 interview reports were collected. The Kano analysis identified 34 community older adult services, with most categorized as attractive or indifferent attributes, and only emergency assistance was identified as a one-dimensional attribute. The BW analysis revealed 3 must-be, 9 attractive, and 1 one-dimensional service. The top five services, based on the Kano-BW-AHP model, were contact with friends and relatives, emergency assistance, regular medical checkups, older adult canteen and meal delivery, and special policy consultation. A comparison between those who chose community-based older adult care and those who did not showed statistically significant differences in six variables: age, education, employee medical insurance, a regular pension, living status, and services awareness (*p* < 0.05). Cluster analysis of participants who chose community older adult care revealed two user profiles: 51 (73.9%) were urban, high-educated users with strong consumption willingness; 18 (26.1%) were rural, low-education individuals with limited income. Community older adult care centers face issues included limited service variety, inadequate beds, insufficient staffing, lack of funding, and no follow-up plans. Additionally, the older adult displayed limited understanding of care services, dissatisfaction with catering, and demand for spiritual support.

**Conclusion:**

The community-based older adult care system shows a structural imbalance between supply and demand, particularly in terms of service quantity, quality, and efficiency. The findings provides a reference for policy formulation and the improvement of community-based older adult care services.

## 1 Introduction

With improvements in healthcare standards, global life expectancy continues to rise (1). Concurrently, the declining fertility rates have become a common trend in global population development (2), leading to the global issue of aging. According to the United Nations’ World Population Prospects 2022, the proportion of the global population aged 65 and older will rise from 10% in 2022 to 16% by 2050. In China, the number of individuals aged 65 and older reached 209.78 million by the end of 2022, accounting for 14.9% of the total population, and is projected to reach 26.1% by 2050 (3), nearing developed-country levels. However, China’s aging process, amid ongoing modernization, reflects both global patterns and distinct national challenges, such as “growing old before getting rich (4)“and “aging before preparation (5)“in certain regions. Consequently, China must adapt its older adult care model rather than simply replicate those of developed countries.

China’s older adult care model comprises home-based, community-based, and institutional care. The key objective of reforming and developing older adult care services is to coordinate these three service forms to create an optimal system (6). Yer the evolving demand structure for older adult exposes imbalances and inadequacies of older adult care service systems: the rise of empty-nest households have rendered home-based care insufficient in meeting the high-quality needs of the older adult, while public health emergencies have highlighted the limitations of institutional care (7). Although studies suggest that the older adult continue to prioritize home care (8–10), social and cognitive factors make institutional care appealing compared to the community -based care (11), revealing a mismatch with service models such as Shanghai’s “90-7-3” and Beijing’s “90-6-4” patterns (12).

In this context, community-based older adult care, with government-enterprise cooperation as its primary operational model, combines the geographical advantages of home care with the specialization of institutional care. As such, it aligns more closely with China’s national conditions (13). Although increasingly recognized as a critical research area, studies on community-based older adult care remain limited (14). Most studies analyze the issue from either the demand or supply side, often from a single perspective (15). In Chongqing, the municipality with the highest aging rate in western China, research on community care for the older adult is limited, typically confined to single district or street (16, 17), with most studies relying on descriptive analysis or traditional qualitative methods (18, 19). Therefore, accurately identifying service demands and strengthening community-based service provision is crucial.

The Kano model, proposed by Professor Noriaki Kano in 1984 (20), explains the non-linear correlation between a product’s performance and user satisfaction. It classifies product attributes into five categories: Attractive, One-dimensional, Must-be, Indifferent and Reverse attributes (21, 22). The Kano model is a simple and effective tool, widely applied across industries for demand classification and prioritization (23–25). Nevertheless, its application in older adult care remains limited, mainly focusing on care demand analysis and application design (26, 27). Given potential inaccuracies in older adult feedback due to cognitive and expression limitations, this study combines hierarchical analysis process with opinions of experts, community-based older adult service managers, grassroots workers and researchers. This approach effectively addresses the limitations of the Kano model in the context of older adult care services. Furthermore, the study incorporates the average satisfaction coefficient as a correction factor in the hierarchical analysis process, reducing the qualitative biases and enhancing the model robustness.

This study is conducted from both supply and demand perspectives, combining quantitative and qualitative analysis to explore supply and demand status of community-based older adult care. The findings aim to provide a decision-making basis for government departments in optimizing the older adult care service system, which will have a profound positive impact on alleviating the imbalance in older adult care service system, improving the well-being of the older adults, and addressing the global aging challenge.

## 2 Materials and methods

### 2.1 Participants

From December 2023 to June 2024, participants were recruited using two-stage sampling in Chongqing, China. In the first stage, one community older adult care center from each of the five-leaf, four-leaf, and three-leaf categories was randomly selected, along with an additional institution to ensure sample representativeness. In the second stage, convenience sampling was employed to conduct an intercept survey of community-dwelling older adults in public spaces within the community, while cluster sampling was used for surveys of older adult residents in care centers. Managers and service users from these three centers were interviewed. A total of 261 questionnaires were received. Two extreme value questionnaires, where all responses were “it does not matter” were excluded. Ultimately, 16 interviews and 259 valid questionnaires were collected, with a 99.2% effective response rate. Inclusion criteria for the older adult participants were: I. ≥ 55 years old; II. ability to answer independently and provide informed consent. Exclusion criteria were: (1) Communication or cognitive impairments; (2) Refusal to participate.

### 2.2 Methods

#### 2.2.1 Questionnaires

The questionnaire comprised two sections: the first part addressed basic demographic information, economic status, and health condition. The second part focused on the supply and demand of community-based older adult care, including understanding of this model, willingness to pay, the preferred care model, and the demand scale for community-based older adult care services based on the Kano model. After a comprehensive review of relevant policies and literature, the five-point Likert scale was developed based on expert consultation and a pre-survey. Service dimensions included life care, medical care, spiritual comfort, culture and entertainment, safety and security, and legal and policy services, with a total of 34 items listed in Table 1. Each item included both positive and negative questions: “How would you feel if this service was provided?” “How would you feel if this service was not offered?” Responses were categorized as “satisfied,” “as it should be,” “it does not matter,” “tolerable,” and “not satisfied.” Reliability and validity was strong, with Cronbach’s alpha for the positive and negative questions were 0.941 and 0.950, respectively, with KMO values of 0.932 and 0.910, respectively, and *p*<0.001.

**Table 1:** Service dimensions and items.

Dimension	Item	Service
Life care	A1	Community canteen or food delivery
	A2	Daily necessities purchases
	A3	Housekeeping
	A4	Handle business such as certification of senior citizens
	A5	Maintenance and installation household equipment
	A6	Bathing aid, haircuts, shaving, etc.
	A7	Daycare provided by community older adult service centers
Medical care	B1	Establishment of health record files
	B2	Smart wearables
	B3	Routine physical examinations
	B4	Accompanying to hospital
	B5	Tele-health services
	B6	Home visit medical services
	B7	Medication reminders
	B8	Health counseling and lectures
	B9	Healthy exercise guidance
	B10	Rehabilitation therapy
	B11	Nutritional guidance
Spiritual comfort	C1	Daily conversations
	C2	Older adult service hotline
	C3	Psychological counselling
	C4	Assistance in contacting relatives and friends
Culture and entertainment	D1	Recreational activities
	D2	Physical exercise
	D3	Interest-based training classes
	D4	Older adult Activity Center
	D5	Group tours for the older adult
	D6	Teaching the use of intelligent products
Safety and security	E1	Emergency rescue
	E2	Safety inspection of the living environment
Legal and policy services	F1	Anti-fraud awareness services
	F2	Legal advice and assistance
	F3	Pension policy consultation
	F4	Special Policy Consultation

#### 2.2.2 Kano model

**Figure 1:**
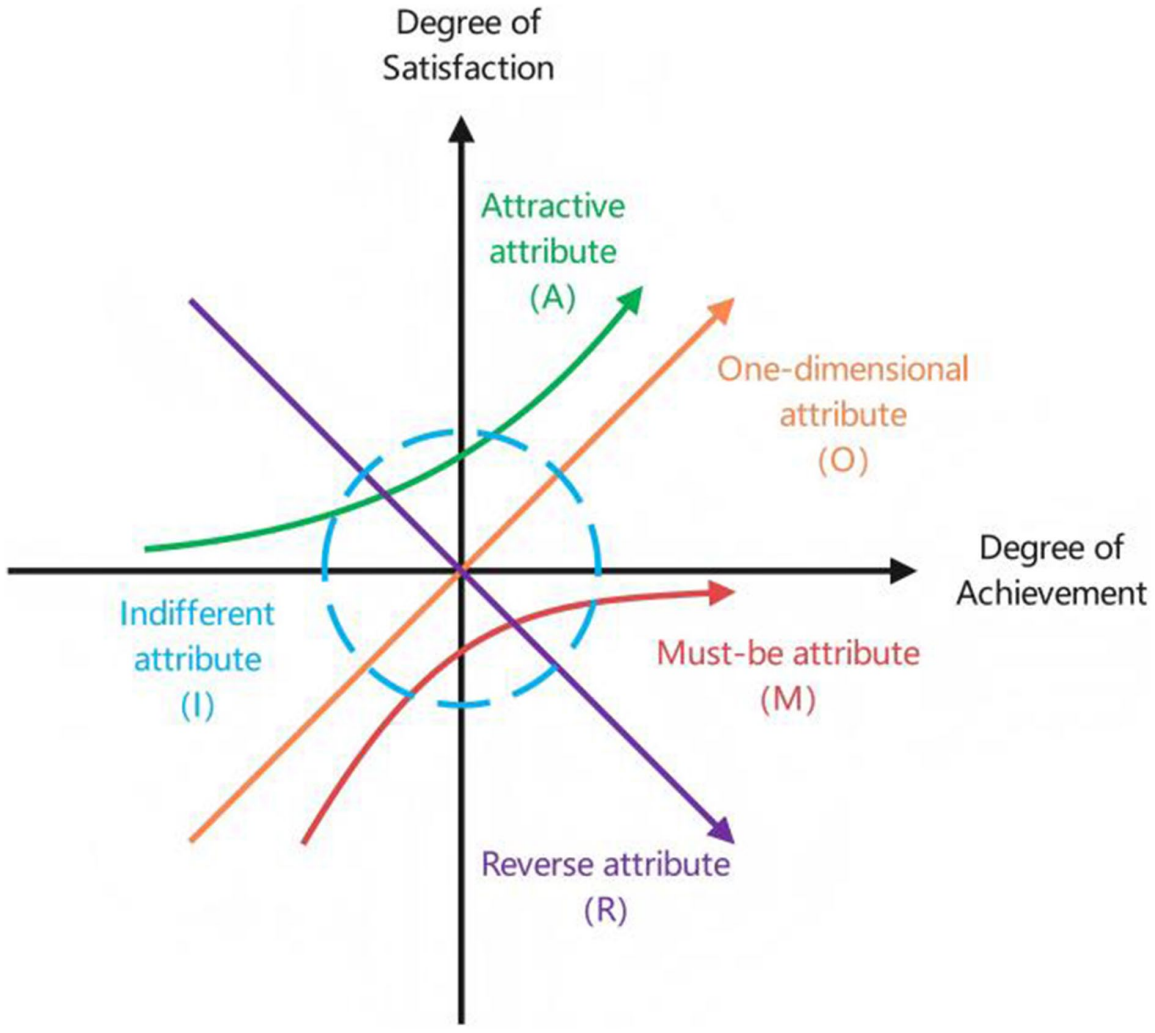
Kano model.

Traditional satisfaction theory posits a linear relationship between product functionality and satisfaction. However, Professor Noriaki Kano challenged this view, proposing the Kano model, inspired by the two-factor theory. According to the model, users have varying levels of demand for product quality, and different elements contribute differently to improving user satisfaction. As shown in Figure 1, must-be attributes do not increase satisfaction when provided, but satisfaction decreases if not provided. Attractive attributes increase satisfaction when provided but do not decrease satisfaction if not provided. One-dimensional attributes have a linear relationship, where satisfaction increases when the attribute performs well, while the satisfaction decreases when the attribute does not perform well. Indifference attributes do not affect the user satisfaction. Reverse attributes lead to a strong user dissatisfaction and should not be provided. Additionally, Questionable attributes, indicating erroneous responses, should be excluded.

#### 2.2.3 Better-Worse (BW) index analysis

As shown in Table 2, users’ responses to positive and negative questions yield 25 possible outcomes, corresponding to different attributes of the Kano model. After determining demand attributes, the Better-Worse index can be calculated.

**Table 2:** Kano model attribute classification table.

Positive questions	Negative questions				
	Satisfied	As it should be	Does not matter	Bearable	Dissatisfied
Satisfied	Q	A	A	A	O
As it should be	R	I	I	I	M
Does not matter	R	I	I	I	M
Bearable	R	I	I	I	M
Dissatisfied	R	R	R	R	Q

M, Must-be attribute; O, One-dimensional attribute; A, Attractive attribute; I, Indifferent attribute; R, Reverse attribute; Q, Questionable attribute.

The Better index is the satisfaction index (SI), and calculated as: Equation 1, which represents the increase in user satisfaction after the service is provided. The closer its value is to 1, the better the satisfaction improvement. The Worse index is the dissatisfaction index (DI), and calculated as: Equation 2, which represents the increase in dissatisfaction when the service is not provided. The closer its absolute value is to 1, the stronger the dissatisfaction effect. The average satisfaction coefficient (ASC) is the arithmetic mean of the absolute values of both indices, and calculated as: Equation 3.


(1) $$\mathrm{SI}=\frac{F(A)+F(O)}{F(A)+F(O)+F(M)+F(I)}$$

(2) $$\mathrm{DI}=-\frac{F(O)+F(M)}{F(A)+F(O)+F(M)+F(I)}$$

(3) $$\mathrm{ASC}=\frac{\mathrm{SI}+|\mathrm{DI}|}{2}$$

#### 2.2.4 Analytic hierarchy process (AHP)

AHP is a hierarchical weighted decision analysis method proposed by Thomas L. Saaty. It treats the decision-making problem as a system, decomposes the objectives into multiple criteria, and then into multiple scenarios, thereby constructing a hierarchical structure model. Subsequently, indicators are compared until a complete judgment matrix is formed (Equation 4). In this study, the 1–5 scale method was used to facilitate understanding and judgment by the older adult.


(4) $$\text{A=}[\begin{array}{cccc} 1 & a_{12} & \cdots & a_{1n}\\ \frac{1}{a_{12}} & 1 & \cdots & a_{2n}\\ \vdots & \vdots & \ddots & \vdots \\ \frac{1}{a_{12}} & \frac{1}{a_{12}} & \cdots & 1 \end{array}]$$

Then, the weight vectors of the matrices were computed using the sum-product method for hierarchical single sorting (Equations 5, 6), and a consistency test was conducted (Equations 7–9).


(5) $$a_{\mathrm{ij}}\prime =\frac{a_{\mathrm{ij}}}{\sum_{i=1}^{n}a_{\mathrm{ij}}}$$

(6) $$w_{i}=\frac{1}{n}\sum_{j=1}^{n}a_{\mathrm{ij}}\prime$$

When the consistency ratio is less than 0.1, the degree of consistency in the judgment matrix is considered acceptable; otherwise, the judgment matrix should be considered.


(7) $$\lambda_{\max}=\frac{\sum {\left(\mathrm{Aw}\right)}_{i}}{{\mathrm{nw}}_{i}}$$

(8) $$\mathrm{CI}=\frac{\lambda_{\max}-n}{n-1}$$

(9) $$\mathrm{CR}=\frac{\mathrm{CI}}{\mathrm{RI}}$$

Finally, a hierarchical ranking can be performed and the decision result can be outputted. In this study, the correction coefficients $\kappa_{i}$ were constructed using normalized ASC to obtain the corrected comprehensive weights (Equation 10).


(10) $$\kappa_{i}=\frac{{\mathrm{ASC}}_{i}}{\sum_{j=1}^{n}{\mathrm{ASC}}_{j}}\times 100$$

### 2.3 Statistics analysis

Data were analyzed using IBM SPSS 26.0. Quantitative variables that met normality assumptions were described as mean ± standard deviation, and *t*-tests were used for between-group comparisons. Frequency and percentages were used to describe categorical and ordinal variables, and *chi*-square tests and Mann–Whitney U tests were applied for between-group comparisons. The Kano-BW-AHP coupling model was employed to categorize and prioritize the attributes of services. The two-step clustering method was utilized to group participants based on their preferences for community-based older adult care. User profile were developed in accordance with the findings of the qualitative analysis.

## 3 Results

### 3.1 General findings

**Figure 2:**
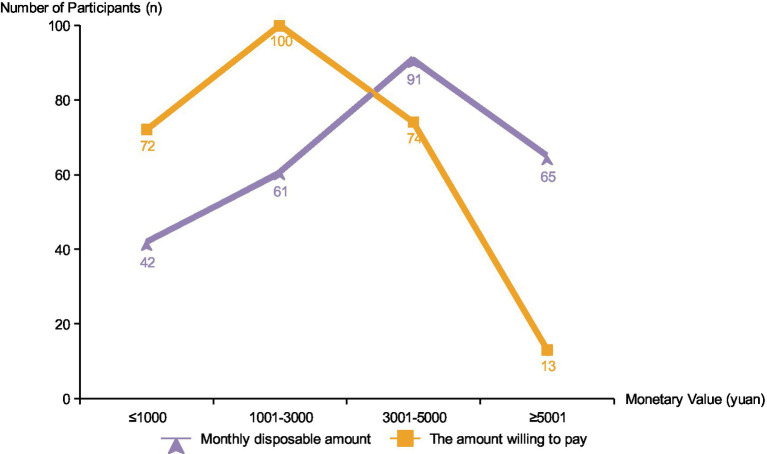
Comparison of disposable income and willingness to pay.

A total of 259 valid questionnaires were collected, with an average age of 78.44± 9.54 years. As shown in Table 3, 11 (4.2%) and 63 (24.3%) participants rated their health status as very poor and poor, respectively, while 106 (41%) participants considered themselves healthy or very healthy. Up to 221 (85.3%) participants had chronic diseases: 85 with one chronic disease, 136 with two or more chronic diseases. When asked about their understanding of the community-based care model, only 12 participants reported knowing it very well, 65 were somewhat familiar, 75 knew a little, and 107 (41.3%) had never heard of it. Regarding their preferred care model, 99 older adult individuals chose traditional institutional care, 91 chose home-based care, and 69 participants preferred community-based care, accounting for 26.6%. Participants’ monthly disposable income and the amount they are willing to pay for community-based older adult care are shown in Figure 2.

**Table 3:** Participants’ conditions related to community-based older adult care (*N* = 259).

Characteristics	Type	Observation (*N*)	Percentage (%)
Self-perceived health condition	Very bad	11	4.2
	Bad	63	24.3
	Normal	79	30.5
	Good	82	31.7
	Very good	24	9.3
Number of chronic diseases	0	38	14.7
	1	85	32.8
	2	74	28.6
	≥3	62	23.9
Self-care capabilities	Completely	203	78.4
	Partially	41	15.8
	Unable	15	5.8
Life satisfaction	Very satisfied	77	29.7
	Relatively satisfied	130	50.2
	Normal	40	15.4
	Relatively dissatisfied	10	3.9
	Very dissatisfied	2	0.8
Level of understanding of community older adult care	Very clear	12	4.6
	Relatively clear	65	25.1
	Heard a little	75	29.0
	Never heard	107	41.3
Preferred older adult care model	Home-based care	91	35.1
	Community-based care	69	26.6
	Institutional-based care	99	38.2

### 3.2 Demand classification based on Kano model

The results of the demand classification were presented in Table 4. None of the 34 services was classified as M, one was designated as O, 20 as A, and 13 as I. In the life care dimension, all services were judged to be A except for A4 handle business, which was judged to be I. In the health care dimension, B2 smart wearables, B5 telehealth services, and B10 rehabilitation therapy were judged to be I, and the rest of them were judged to be A. While the services in the spiritual comfort and legal policy dimensions were mostly judged to be I, except for C1 daily chat and F1 anti-fraud promotion services, which were A.

**Table 4:** Frequency of demand for community-based older adult care services.

Item	Frequency						Results
	M	O	A	I	R	Q	
A1	31	82	95	47	2	2	A
A2	10	36	118	89	5	1	A
A3	19	56	114	68	1	1	A
A4	10	30	102	114	0	3	I
A5	14	46	128	66	3	2	A
A6	21	55	98	81	1	3	A
A7	15	44	103	91	1	5	A
B1	16	41	104	98	0	0	A
B2	3	18	108	122	5	3	I
B3	29	72	103	52	1	2	A
B4	16	50	106	80	3	4	A
B5	1	24	104	127	2	1	I
B6	9	47	123	75	2	3	A
B7	8	46	102	98	3	2	A
B8	8	41	115	92	1	2	A
B9	7	29	125	94	3	1	A
B10	9	32	106	110	1	1	I
B11	6	28	119	103	3	0	A
C1	8	35	126	86	3	1	A
C2	13	35	105	106	0	0	I
C3	13	17	100	127	2	0	I
C4	32	61	66	96	3	1	I
D1	16	65	106	68	3	1	A
D2	10	45	108	96	0	0	A
D3	8	48	121	80	2	0	A
D4	20	58	112	67	1	1	A
D5	2	21	89	141	5	1	I
D6	6	22	107	120	3	1	I
E1	40	115	55	47	1	1	O
E2	20	46	92	96	3	2	I
F1	9	36	114	96	2	2	A
F2	5	19	95	137	3	0	I
F3	17	31	92	114	4	1	I
F4	9	62	81	102	4	1	I

M, Must-be attribute; O, One-dimensional attribute; A, Attractive attribute; I, Indifferent attribute; R, Reverse attribute; Q, Questionable attribute.

### 3.3 The Better-Worse index analysis

**Figure 3:**
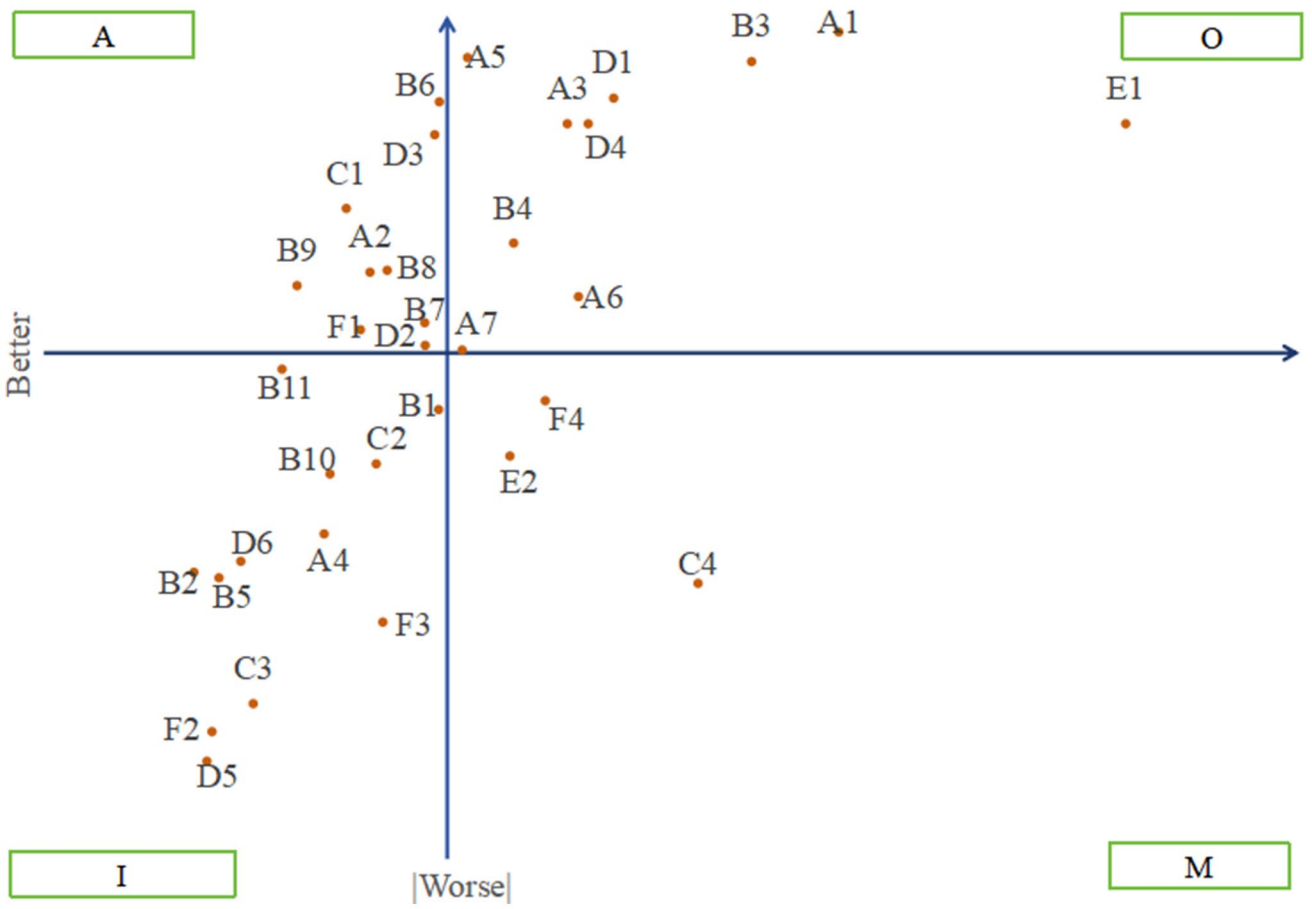
Four-quadrant scatterplot of community-based older adult care services.

As shown in Figure 3, the average value of the Better-Worse index of 34 services (0.225, 0.580) was used as the origin of coordinate system, with the *Y*-axis representing the Better value and the X-axis representing the absolute value of the Worse. A four-quadrant chart was constructed, and from the first quadrant to the fourth quadrant represents O, A, I, and M, respectively. Among these, the ASC is the highest in the first quadrant, with E1 emergency rescue having the highest ASC, followed by A1 community canteen or food delivery, B3 routine physical examination. In the second quadrant, the ASC of B6 home visit medical services and B4 accompanying to hospital was higher. The third quadrant includes B1 health records, C2 older adult service hotline, B11 nutrition guidance. The ASC in the fourth quadrant, from high to low, is C4 assistance in contacting relatives and friends, F4 special policy consultation, and E2 safety inspection of the living environment.

### 3.4 Results of the analytic hierarchy process

Based on the results of the Better-worse index analysis, the indifferent attribute in the third quadrant was eliminated, and the must-be, one-dimensional, and attractive attributes were output as the standard-level indicators of the AHP model. The service items under each attribute were used as the scheme-level indicators. Judgment matrices were constructed, and experts were invited to compare and score multiple indicators pairwise. A total of 10 individuals were invited to score: four managers from older adult care service centers, three knowledgeable older adult individuals, two staff members who participated in the survey throughout the process, and one industry expert. All integrated matrices underwent consistency testing, with CR<0.1, validating the results. The results of the hierarchical single sorting are presented in Table 5.

**Table 5:** Results of the hierarchical single sorting.

Criterion layer	weight coefficient	Consistency inspection results	Indicator layer	weight coefficient	Consistency inspection results
M	0.287	$\lambda_{\max}$ =3.022 CI = 0.011 CR = 0.021	C4	0.594	$\lambda_{\max}$ =3.004
			F4	0.287	CI = 0.002
			E2	0.119	CR = 0.004
O	0.594		E1	0.158	$\lambda_{\max}$ =10.138 CI = 0.015 CR = 0.010
			A1	0.115	
			B3	0.138	
			D1	0.091	
			D4	0.088	
			A3	0.075	
			A5	0.071	
			A6	0.077	
			D3	0.090	
			A7	0.098	
A	0.119		B6	0.193	$\lambda_{\max}$ =9.045 CI = 0.006 CR = 0.004
			B4	0.176	
			D2	0.074	
			B8	0.098	
			C1	0.093	
			B7	0.105	
			A2	0.091	
			F1	0.092	
			B9	0.077	

### 3.5 Ranking results based on coupling model

The hierarchical total sorting results and the corrected comprehensive weights are shown in Table 6. The top five services are C4 Contacting Relatives and Friends, E1 Emergency Rescue, B3 Regular Physical Examination, A1 Canteen or Meal Delivery, and F4 Special Policy Consultation. Conversely, the bottom five services are F1 Anti-fraud Publicity, B9 Health Exercise Guidance, and D2 Sports Fitness. After the correction, significant changes occurred in the ranking of C4 Contacting Relatives and Friends and D2 Sports Fitness. The corrected rankings were further confirmed through the questionnaire survey process and in-depth interviews with the older adult, demonstrating their authentic and effective.

**Table 6:** Hierarchical total sorting and the comprehensive weights.

Item	Comprehensive weights	Correction coefficient	Corrected weight	Ranking
C4	0.170	4.374	0.746	1
E1	0.094	6.412	0.601	2
B3	0.082	5.464	0.446	3
A1	0.068	5.763	0.393	4
F4	0.082	4.273	0.352	5
D1	0.054	5.008	0.271	6
D4	0.052	4.891	0.255	7
A7	0.058	4.126	0.241	8
D5	0.053	4.435	0.236	9
A3	0.044	4.830	0.215	10
A6	0.046	4.551	0.208	11
A5	0.042	4.668	0.198	12
E2	0.034	4.070	0.139	13
B6	0.023	4.511	0.104	14
B4	0.021	4.465	0.094	15
B7	0.012	4.029	0.050	16
B8	0.012	4.060	0.047	17
C1	0.011	4.055	0.045	18
A2	0.011	4.009	0.044	19
F1	0.011	3.877	0.043	20
B9	0.009	4.060	0.037	21
D2	0.009	4.070	0.036	22

### 3.6 User profile based on cluster analysis

**Figure 4:**
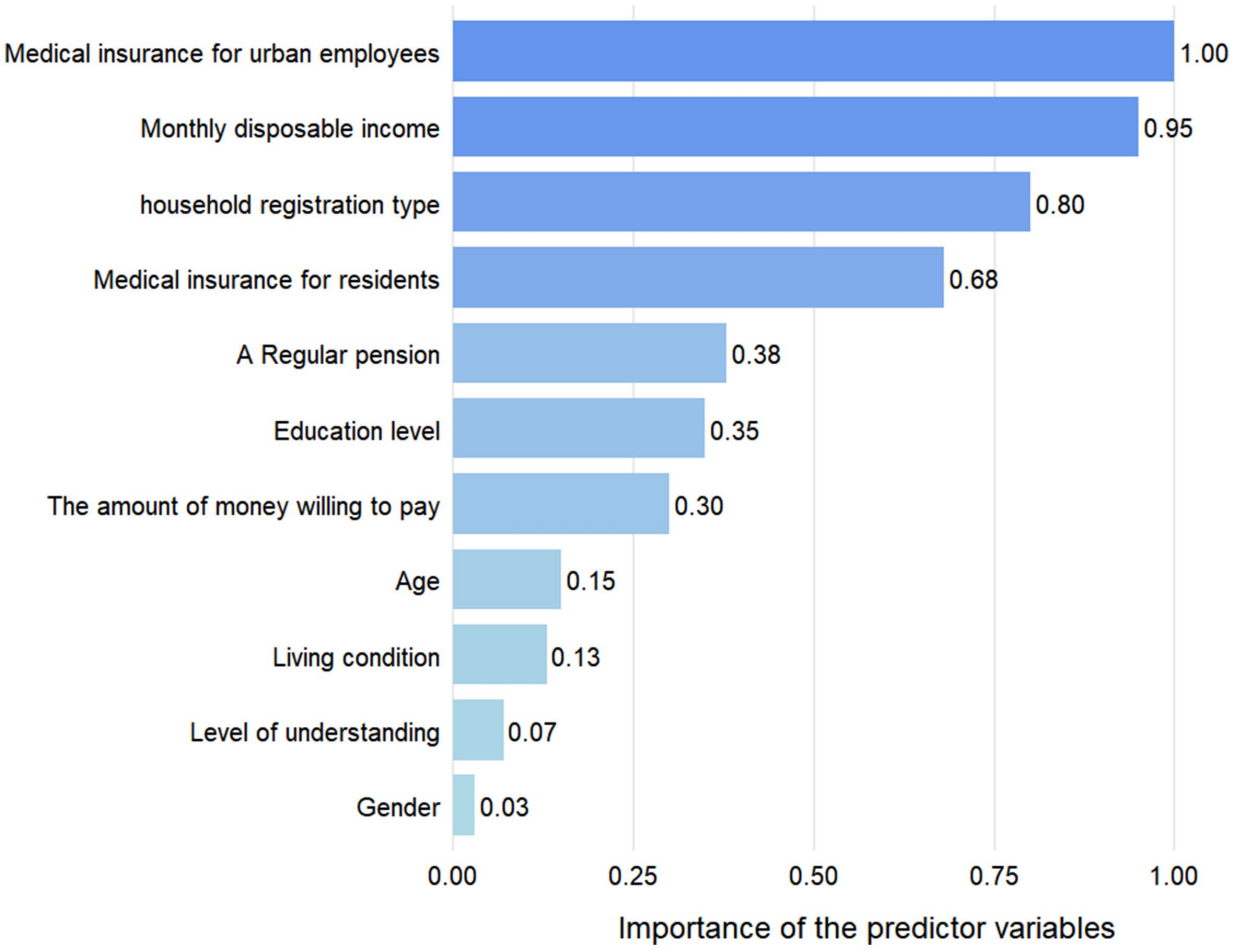
The predictor materiality.

The mean age of participants in the group selecting community-based older adult care (76.09 ± 9.76) was significantly lower than the group choosing other options (79.29 ± 9.4), *t* = 2.42, *p*<0.05. The results of the univariate analysis are shown in Table 7. Between the two groups of participants, whether they community-based older adult care, the differences in age, education level, employee medical insurance, a regular pension, living situation, and understanding of community-based care were all statistically significant (*p*<0.05). After comprehensive consideration, the aforementioned six variables, along with three fundamental variables—gender, household registration type, and resident medical insurance—and two economic variables, namely, the amount of monthly disposable income and the amount of money willing to pay for community-based older adult care, were included in the two-step cluster analysis. The clustering results of user portraits identified two categories, and the importance of the predictor variables is shown in Figure 4.

**Table 7:** Univariate analysis of whether to choose community-based care for the older adult.

Characteristics	Type	Community-based older adult care	Others	*χ^2^/U*	*p*
Gender	Female	42	102	1.059	0.304
	Male	27	88		
Household registration	Urban	52	128	1.526	0.217
	Rural	17	62		
Marital status	Unmarried	4	14	4.823	0.178
	Married	37	89		
	Divorced	4	3		
	Widowed	24	84		
Living condition	Living alone	18	43	10.624	0.031*
	With spouse	31	71		
	With descendants	6	50		
	With spouse and descendants	8	11		
	Other situations	6	15		
Medical insurance for urban employees	Yes	50	103	6.976	0.008**
	No	19	87		
Medical insurance for urban and rural residents	Yes	14	56	2.165	0.141
	No	55	134		
Alimony given by offspring	Yes	31	67	2.010	0.156
	No	38	123		
A regular pension	Yes	59	138	4.609	0.032*
	No	10	52		
Education level	Uneducated	5	35	5137.000	0.006**
	Primary school	19	67		
	Junior high school	17	25		
	Senior high school	11	39		
	Junior college or higher	17	24		
Number of offspring	0	6	19	5628.500	0.067
	1	17	31		
	2	29	65		
	≥3	17	75		
Self-perceived health condition	Very bad	4	7	6340.500	0.676
	Bad	18	45		
	Normal	18	61		
	Good	24	58		
	Very good	5	19		
Self-care ability	Completely	55	148	5921.500	0.157
	Partially	13	28		
	Unable	1	14		
Life satisfaction	Very satisfied	20	57	6228.500	0.505
	Relatively satisfied	39	91		
	Normal	9	31		
	Relatively dissatisfied	0	10		
	Very dissatisfied	1	1		
Degree of understanding	Very clear	4	8	4974.000	0.002**
	Relatively clear	25	40		
	Heard a little	22	53		
	Never heard	18	89		
Monthly disposable amount (yuan)	≤1,000	5	37	6463.500	0.858
	1,001–3,000	21	40		
	3,001–5,000	30	61		
	≥5,000	13	52		
The amount willing to pay (yuan)	≤1,000	11	61	6191.500	0.472
	1,001–3,000	39	61		
	3,001–5,000	15	59		
	≥5,000	4	9		

The asterisk denotes statistically significant differences *p < 0.05; **p < 0.01; ***p < 0.001.

In category 1, there were 51 (73.9%) participants with a mean age of 77.5 years. Of these, 47 (92.2%) were urban residents with employee medical insurance; 43 (84.3%) participants had a monthly disposable income exceeding 3,000 yuan; 48 (94.1%) received a regular pension; 28 (54.9%) had a high school education or higher, and only 1 participant was uneducated; 28 (54.9%) were willing to pay 1,001–3,000 yuan per month for community-based care, while 19 (28%) were willing to pay over 3,000 yuan per month. In category 2, there were 18 (26.1%) participants with a mean age of 72.1 years. Of these, 11 (61.1%) had medical insurance for urban and rural residents; 13 (72.2%) had a monthly disposable income of 1,001–3,000 yuan, while the rest had less than 1,000 yuan; 13 (72.2%) held a rural household registration; 11 (61.1%) received a regular pension; 14 (77.8%) had completed primary school or junior high school, while the remaining participants being uneducated; 11 (61.1%) were willing to pay 1,001–3,000 yuan for community-based care, while the remaining 7 (38.9%) were willing to pay less than 1,000 yuan.

The results yielded two types of user profiles: the urban, high-educated group with favorable economic conditions and strong consumption willingness, and the rural, low-education group with limited income and weak ability to pay.

### 3.7 Qualitative analysis results

The three centers faced five common operational problems: I. The service forms were predominantly long-term care and daycare, with home visit services less frequently conducted. II. The centers had 118/90/28 beds respectively, and almost all of them were occupied. III. Inadequate staffing was a significant concern, 40/14/12 staff members respectively, leading to situations where one person worked across multiple departments. Occasional services by volunteers from social organizations or schools were mainly focused on spiritual recreational activities. IV. Government subsidies were insufficient, and the centers were primarily financed by the older adult at their own expense. V. The primary services provided were medical care, life care, and spiritual entertainment, with canteens being the most commonly utilized services while free entertainment services were the most popular. Notably, all three centers indicated that they had no follow-up plans.

Interviews with the older adult also revealed four common problems: (1) 12 older adult interviewees had a low level of understanding of community-based older adult care, with their primary sources of information being TV, WeChat posts, and conversations with relatives and friends. Only 1 person had consulted the service center offline for a systematic understanding; (2) Regarding existing services, 7 respondents mentioned that the quality of catering services was suboptimal, 1 respondent suggested enhancements to the medical technology, and 1 proposed shorter inspection intervals. (3) When queried about prospective service utilization, two respondents expressed a need for assistance in contacting relatives and friends, while another expressed a desire for long-term companionship services for individuals with prolonged bedridden status. (4) Older adult people with impaired self-care ability believed that the quality of long-term custodial services would not be good, and therefore, they still preferred home-based services.

## 4 Discussion

### 4.1 Summary

Based on 259 valid questionnaires and 16 interviews for both the supply and demand sides of community older adult care, this study systematically analyzed the demand attributes and priorities of 34 community older adult services using the Kano-BW-AHP coupled model. Two types of user profiles were identified through two-step clustering, and supply–demand contradictions were summarized through qualitative analysis, revealing the current structural imbalance between supply and demand for community-based older adult care services in western China.

### 4.2 Discussion

In line with the existing research (26–29), 69 (26.6%) older adult participants prefer community-based older adult care services, and 182 (70.3%) participants knew little about them. In contrast, findings from a study in Beijing were quite different, with more than 75% of older adults having used community-based aging services or having some knowledge of them, and over 80% expressing a strong willingness to use community-based aging service facilities (30). This spatial heterogeneity suggests that the deepening implementation of community-based older adult services in the western China faces the dual dilemmas of perception and choice. Therefore, the government, media, and community should enhance the systematic publicity of community-based older adult care services, with age-appropriate messaging.

In the Kano analysis, no service was judged as M, and only 1 service was judged as O. Researchers observed that Chinese older adult individuals tend to be subtle and easy-going, often not expressing dissatisfaction. Thus, re-identifying needs through BW analysis was necessary and effective. The Kano-BW analysis reclassified 3 services as M, 9 as O and 10 as A. This study also introduces empirical opinions from experts and managers through AHP, constructing correction coefficients using the original data of the preorder model, which ultimately forms the Kano-BW-AHP model. After correction, the rankings of helping to contact relatives and friends and special policy counseling increased significantly, which was confirmed in interviews, validating the applicability and effectiveness of the model.

Through Kano-BW-AHP analysis, the top five services in the demand ranking are various dimensions of contacting relatives and friends, emergency assistance, regular medical checkups, canteen or meal delivery, and special policy consultation. This indicates that the overall demand for community older adult services is multidimensional, suggesting the need for service providers to establish a multi-level structure with hierarchical demand levels. Furthermore, it is apparent from these demand dimensions that older adults have a heightened need for safety. A study in Helsinki (31) found that loneliness, limited social networks, and social age discrimination were sources of insecurity among home-dwelling older adults. Studies in China have shown that the sense of security among home-dwelling older adult individuals is linked to parent–child support, community environment, and social care (32, 33). Therefore, community-based older adult services should strengthen the sense of security for the older adult in all aspects, enhance community service promotion with family participation and support, modify the community environment to be age-friendly, and foster a positive community atmosphere for the older adult.

The study found that the older adult have a high prevalence of chronic diseases (85.3%), and the comorbidity of chronic diseases was common (52.5%). Similar to the previous findings (34), services under the healthcare dimension, such as regular medical checkups and home visit medical services, remain the focus of older adult individuals’ attention. Efforts should be made to improve the quality and efficiency of healthcare services and enhance accessibility for the older adult. On the other hand, the demand for smart wearable devices and telemedicine was not high, which aligns with the low willingness of the older adult to use smart medical services, as noted in relevant studies (35). Some studies have identified subjective and objective difficulties in the acceptance and use of smart devices by the older adult (36). In the digital information age, a more reliable user-friendly smart healthcare service environment should be created for the older adult.

Previous studies (37) have demonstrated that recreational activities are significantly related to the mental health and well-being of the older adult. The interviewed older adult also expressed their demand for recreational activities and senior activity centers, which they ranked 6th and 7th, respectively. It is thus essential to ensure the availability of activity venues and diversify the types of activities offered. Nevertheless, fitness exercise ranked last. This disparity may stem from factors related to physical condition and inherent beliefs. It is recommended that relevant institutions promote recreational activities with fitness benefits, such as dancing, and card games, and strengthen health education efforts.

However, older adult individuals with disabilities placed more importance on life care-related services, which were predominantly categorized as A in this study. The difficulty of home-based care and the shortage of institutional-based care personnel are urgent practical problems that need addressing. The pressure of aging on society and families should be alleviated through various approaches, such as strengthening nursing professional training (38), developing auxiliary care equipment like multi-functional nursing beds (39) and household mobility devices (40), and encouraging community residents to join time banks for mutual older adult assistance (41).

The continued development of home visit services should be a priority for policymakers, as previous studies (42) have indicated that home visits are one of the unmet community-based care services for older people. The results of this study reveal that emergency assistance, living environment safety inspection, home delivery, and home medical care are services urgently needed by the older adult. The development of community mutual care model is a promising strategy to meet this demand amidst a shortage of human resource (27).

Finally, two types of user profiles through cluster analysis. On the one hand, urban high-educated users (73.9%) with high income and a strong willingness to pay are the primary consumers of the silver-hair economy, and their diverse demands need to be met through service quality improvements to promote the high-quality development of community-based older adult care services. On the other hand, the pilot reform of community older adult care has been particularly effective in promoting consumption and improve the welfare of low-age older adult, low-income older adult families, and rural older adult individuals (43), which comprise the second category of users (26.1%) in this study. This group requires policy support and basic services to improve service accessibility and unlock the payment potential of these groups. Additionally, the differences in the profiles and proportions of these two user types indicate a structural imbalance in the supply and demand for community-based older adult care services.

At the community older adult care service system is fully implemented, relevant departments should continue to empower the system, with particular focus on talent training, publicity and education, financial guarantee, and policy support to address the current imbalance in community-based older adult care services.

### 4.3 Limitation

This study has certain limitations. Firstly, potential bias may arise from the small sample size and the use of convenience sampling in the two-stage sampling process. Secondly, due to the older age of those who voluntarily participated in the survey on older adult service demand and supply, the demand of younger older adult individuals for community-based older adult services was not explored, and some older adult individuals refused to participate due to hearing problems, which affected the generalizability of the findings. Finally, the cross-sectional data of the current survey are used, and the analysis results are relatively limited. In the future, these problems will be supplemented and solved, and the research on community-based older adult care services will be further improved.

## 5 Conclusion

This study comprehensively evaluates the status of community-based older adult care services from both supply and demand perspectives. By applying the Kano-BW-AHP model, the demand attributes of each service were distinguished and ranked, providing a quantitative analytical framework to accurately identify the older adult’s demands. Through clustered user profiling and qualitative analysis, the characteristics of two types of users who are likely to choose community-based older adult care were summarized, common problems requiring resolution by both supply and demand were discussed, and the study reveals an imbalance between the supply and demand of community-based older adult care services in terms of quantity, quality, efficiency, and structure. The findings can serve as a reference for policymakers, practitioners, and researchers, providing a basis for optimizing resource allocation, accurately supplying multidimensional services, and improving the imbalance between supply and demand.

## Data Availability

The raw data supporting the conclusions of this article will be made available by the authors, without undue reservation.
